# Adverse fetal outcomes and its associated factors in Ethiopia: a systematic review and meta-analysis

**DOI:** 10.1186/s12887-020-02176-9

**Published:** 2020-06-03

**Authors:** Getnet Gedefaw, Birhan Alemnew, Asmamaw Demis

**Affiliations:** 1 Department of Midwifery, College of health sciences, Woldia University, Woldia, Ethiopia; 2 Department of Medical laboratory science, College of health sciences, Woldia University, Woldia, Ethiopia; 3 Department of Nursing, College of health sciences, Woldia University, Woldia, Ethiopia

**Keywords:** Meta-analysis, Neonatal outcomes, Systematic review

## Abstract

**Background:**

Despite the reduction of neonatal morbidity and mortality, is one of the third Sustainable Development Goal to end the death of children, the burden of the problem still the major challenge in Ethiopia. Globally, the most common causes of neonatal morbidity and mortality are adverse fetal outcomes (low birth weight, stillbirth, prematurity, congenital defect). Therefore this systematic review and meta-analysis aimed to estimate the pooled prevalence of adverse fetal outcomes and its associated factors in Ethiopia.

**Method:**

International databases (PubMed, Google scholar, web of science and science direct) were searched. Seventeen articles were included, among these, fourteen were cross-sectional and three of them were case-control studies. Publication bias was employed using a funnel plot and eggers test. The I^2^ statistic was computed to check the heterogeneity of studies. Subgroup analysis was performed for the evidence of heterogeneity.

**Result:**

A total of 11,280 study participants were used to estimate the pooled prevalence of adverse fetal outcomes. The overall pooled prevalence of adverse fetal outcomes in Ethiopia was 26.88% (95% CI; 20.73–33.04). Low birth weight 10.06% (95% CI; 7.21–12.91) and prematurity 8.76% (95% CI; 5.4–12.11) were the most common adverse birth outcome at the national level. Rural in residency (AOR = 2.31; 95% CI: 1.64–3.24), lack of antenatal care follow up (AOR = 3.84; 95% CI: 2.76–5.35), pregnancy-induced hypertension (AOR = 7.27; 95% CI: 3.95–13.39), advanced maternal age ≥ 35(AOR = 2.72; 95% CI: 1.62–4.58, and having current complication of pregnancy (AOR = 4.98; 95% CI: 2.24–11.07) were the factors associated with adverse birth outcome.

**Conclusion:**

The pooled prevalence of adverse fetal outcomes in Ethiopia was high. Rural in residency, lack of antenatal care follow up, pregnancy-induced hypertension, advanced maternal age ≥ 35, and having current complications of pregnancy were the factors associated with adverse fetal outcomes.

**PROSPERO protocol registration:**

CRD42020149163.

## Background

Adverse fetal outcome is the major challenge both in low and middle-income countries. Globally, adverse birth outcomes such as preterm birth, low birth weight, stillbirth, and congenital defect are some of the common problems. Neonatal morbidities and mortalities are one of the most common contributing factors for 11.8 million deaths. Even though neonatal mortality is declined globally, highest in sub-Saharan Africa and South Asia, with each estimated at 27 deaths per 1000 live births in 2017 [1].

Low birth weight is one of the most common causes of neonatal morbidity and mortality worldwide. Globally, low birth weight (LBW) is one of the major neonatal problems that predispose neonates to different neonatal complications, such as hypoglycemia, hypothermia, and different acute and long-term developmental complications [2–6]. Epidemiologically, 15 to 20% of newborns are low birth weight globally; among this 4.53% of them are accounted in Ethiopia [7, 8].

Every year, more than 7 million perinatal deaths occur across the world, and half of them are stillbirth’s accounts for 3.5 million stillbirths. The rate of stillbirth in developed countries is estimated between 4.2 and 6.8 per 1000 births whereas in low and middle-income countries ranges from 20 to 32 per 1000 births [9–11]. In sub-Saharan Africa, more than 900,000 babies die as stillbirths [12]. Among sub-Saharan African countries, Ethiopia is a country where the highest proportion of stillbirths has occurred. According to the systematic review done from 1974 to 2013 in Ethiopia showed that the magnitude of stillbirths is 60–110 /1000 births [13].

Prematurity is another important risk factor for neonatal complications. Each year estimated 13 million newborns born before 37 weeks of gestation which contributes to 27% of neonatal deaths; in the world; meaning more than one million preterm babies die each year due to prematurity [14]. Despite the institutional delivery and antenatal care follow up is increasing rapidly still, neonatal death is increasing.

Worldwide, over 303,000 newborns die within 4 weeks of birth every due to congenital anomalies. Congenital anomalies can contribute to long-term disability, which may have significant impacts on individuals, families, and societies. The most common, severe congenital anomalies are heart defects, spinal Bifida, anencephaly, severe hydrocephalus, neural tube defects and Down syndrome [15].

According to 2019 EMDHS, neonatal mortality is increasing to 30 /1000 births as compared to 2016 EDHS showed that 29/1000births. Therefore, this systematic review and meta-analysis are aimed to estimate the overall prevalence of adverse birth outcomes (low birth weight, preterm birth, stillbirth, and congenital defect) and secondly identify factors contributing to adverse birth outcomes in Ethiopia.

## Methods

This systematic review and meta-analysis were conducted to estimate the pooled prevalence of adverse fetal outcomes, the most common magnitude of adverse fetal outcomes and associated factors in Ethiopia using the standard PRISMA checklist guideline.

### Searching strategy

International databases (Pub Med, Google Scholar, Web of Science), different gray pieces of literature and articles published in the university online repository were included. Core searching terms were used using PICO formulating questions. These were: “newborn”, “adverse birth outcome”, “fetal outcome”, “stillbirth”, “low birth weight”, “neonate”, “prematurity”, “congenital anomaly”, “congenital defect”, “preterm”, “preterm birth”. “Ethiopia”. The following Searching terms were applied: neonate OR newborn OR women OR infant OR child OR children AND “abnormal birth weight” OR “congenital defect” OR “congenital anomaly” OR “stillbirth” OR “prematurity” OR “preterm birth” OR “low birth weight” OR “perinatal” OR “neonatal death” OR “preterm “AND Ethiopia and related terms. The search strategy has been employed from July 3/2019- September 30/2019.

### Inclusion and exclusion criteria

Observational studies (case-control and cross-sectional) were included. Articles reported the prevalence or/ and a minimum of one contributing factor for adverse fetal outcomes is included. Only English language literature and research articles were included. Studies reported overall adverse fetal outcomes and/or associated factors were included. Whereas, articles without full abstracts or texts and articles reported out of the outcome interest were excluded.

### Quality assessment

Two authors (GG & AD) independently assessed the quality of each study using the Joanna Briggs Institute (JBI) quality appraisal checklist was used [16]. Any disagreement was resolved by the hindrance of the third reviewer (BA). The following JBI items used to appraise case-control studies were: [1] comparable groups, [2] appropriateness of cases and controls, [3] criteria to identify cases and controls, [4] standard measurement of exposure, [5] similarity in the measurement of exposure for cases and controls, [6] handling of confounder [7], strategies to handle confounder, [8] standard assessment of outcome, [9] appropriateness of duration for exposure, and [10] appropriateness of statistical analysis. Items used to appraise cross-sectional studies are: [1] inclusion criteria, [2] description of study subject and setting, [3] valid and reliable measurement of exposure, [4] objective and standard criteria used, [5] identification of confounder, [6] strategies to handle confounder, [7] outcome measurement, and [8] appropriate statistical analysis. Therefore to consider the studies have low risk, the value should be 50% and above the quality assessment indicators.

### Data extraction

After collecting findings from the entire database, the articles were transferred from Endnote version X8 software to the Microsoft Excel spreadsheet to remove duplicated studies. Two authors (AD and GG) independently extracted all the important data using a standardized JBI data extraction format. Any disagreement between reviewers was resolved by the third reviewer (BA) through discussion and consensus. The name of the author, sample size, publication year, study area, response rate region, the overall prevalence of adverse fetal outcome with its outcome categories with 95%CI and associated factors were collected.

### Outcome of measurements

**Adverse fetal outcome**; at least one of the following (stillbirth, low birth weight, preterm and congenital anomaly) was reported.

**Stillbirth** is the death of the newborn after 28 weeks of gestation and during labor.

**Preterm birth/prematurity**: as having a Gestational age at the birth of < 37 weeks.

**Congenital anomaly**: was considered, when newborn recorded having any body parts of congenital defects.

**Low birth weight**: was considered, when newborn weight recorded below 2500 g. Moreover, the outcome of this study extends to identify associated factors of adverse birth outcomes.

### Data analysis

Publication bias was checked using the funnel plot and Egger’s regression test [17]. The heterogeneity of studies was computed using the Cochrane Q-test and I-squared statistic [18, 19]. Pooled analysis was conducted using a weighted inverse variance random-effects model [20]. Subgroup analysis was conducted using the study region and year of publication. STATA version 11 statistical software was used. Forest plot format was used to present the pooled point prevalence with 95%Cl. For associations, a log odds ratio was used to decide the association between associated factors and adverse fetal outcomes.

## Result

### Characteristics of the included studies

347 articles were retrieved using a search strategy regarding adverse fetal outcomes and associated factors in Ethiopia at PubMed, Google Scholar, Science Direct, a web of science and other gray literature. After duplicates were expunged, 245 studies remained.

**Fig. 1:**
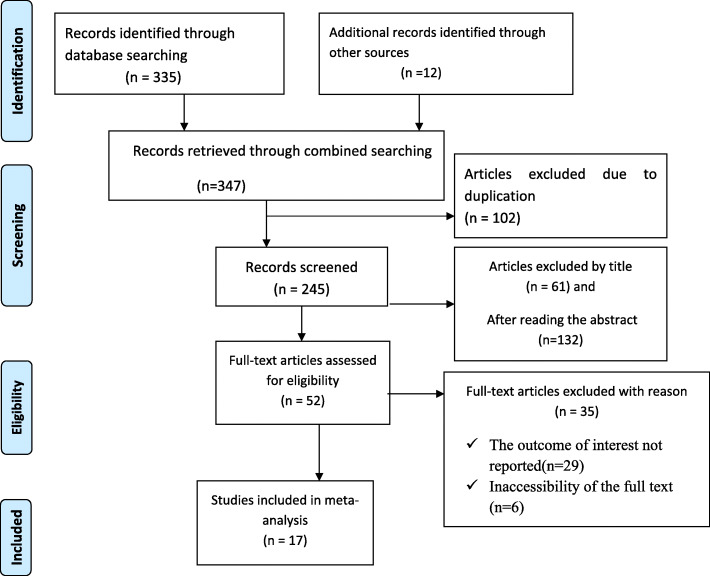
Flow chart of study selection for systematic review and meta-analysis of adverse fetal outcomes and its associated factors in Ethiopia

Out of the remaining 245 articles, 193 articles were excluded after review of their titles and abstracts. Therefore, 52 full-text articles were accessed and assessed for inclusion criteria, which resulted in the further exclusion of 35 articles primarily due to reason. As a result, 17 studies were met the inclusion criteria to undergo the final systematic review and meta-analysis **(**Fig. 1**).**

This review is also included factors contributing to adverse neonatal/fetal outcomes; categorized as socio-demographic factors (maternal age, monthly income, residence), obstetric and medical-related factors (pregnancy complication during the current pregnancy, parity, gravidity, status of antenatal care follow up, pregnancy-induced hypertension, antepartum hemorrhage, anemia, multiple pregnancies, bad obstetric history, and rupture of membrane) are the factors associated with adverse pregnancy outcomes [21–36].

All the included studies were conducted from different regions of Ethiopia (Amhara, Oromia, SNNPR (South Nation Nationalities people and representatives), Tigray, Somali and Addis Ababa. Finally, this systematic review and meta-analysis consist of seventeen articles: fourteen studies were cross-sectional and three of them were case-control with a total study participant of 11,280 infants. In this table, the outcome of interest, the number of study participants, prevalence and response rate of the original studies were included. The maximum and minimum sample size amongst the included studies was reported in the Amhara region with a population of 3003 and 295 at Bahirdar and North Wollo respectively **(**Table 1**).**

**Table 1:** Study characteristics included in the systematic review and meta-analysis

Authors	Region	Study area	Study design	Sample size	Prevalence	Response rate	Outcome variables	Quality
Abebe Eyowas et al. [21]	Amhara	Bahirdar	cross sectional	3003	37.796	100%	Preterm birth, LBW, Stillbirth	Low risk
Ritbano A et al. [22]	SNNPR	Bitajira	cross sectional	313	18.211	100%	Preterm birth, LBW, Stillbirth & congenital defect	Low risk
Kebede et al. [23]	Amhara	D/tabor	case control	620	–	100%	–	Low risk
Cherie N, Mebratu A [24]	Amhara	Dessie	cross sectional	462	32.468	100%	Preterm birth, LBW, Stillbirth & congenital defect	Low risk
Kassa et al. [25]	Amhara	E/gojjam	cross sectional	1134	19.224	90.40%	Preterm birth, LBW, Stillbirth	Low risk
Feleke G et al. [26]	SNNPR	Gamo gofa	case control	420	–	98.50%	–	Low risk
Adane et al. [27]	Amhara	Gondar	cross sectional	481	22.661	98.16%	Preterm birth, LBW, Stillbirth	Low risk
Tsegaye and Kassa [28]	SNNPR	Hawassa	cross sectional	580	18.276	100%	Preterm birth, LBW, Stillbirth & congenital defect	Low risk
Abdo et al. [29]	SNNPR	Hossana	cross sectional	327	24.465	100%	Preterm birth, LBW, Stillbirth & congenital defect	Low risk
Eyosias Yeshialem [30]	Oromia	Jimma	case control	344	–	100%	–	Low risk
Eshete A et al. [31]	Amhara	North wollo	cross sectional	295	23.051	100%	Preterm birth, LBW, Stillbirth & congenital defect	Low risk
Ediris et al. [32]	Oromia	Shashemene	cross sectional	306	34.967	100%	Preterm birth, LBW, Stillbirth & congenital defect	Low risk
Abera Haftu et al. [33]	Tigray	Shire	cross sectional	425	22.588	100	Preterm birth, LBW, Stillbirth	Low risk
Tsegaye Lolaso [34]	SNNPR	Kembata	cross sectional	718	13.928	93%	Preterm birth,LBW, Stillbirth	Low risk
Mekonnen M, et al. [37]	Somali	Fafan	cross sectional	1050	51.905	98.30%	Stillbirth, congenital defect	Low risk
Hailemariam Workie [35]	Tigray	Mekelle	Cross sectional	340	25	100%	Preterm birth,LBW, Stillbirth	Low risk
kassahun et al. [36]	Amhara	Woldia	cross sectional	462	31.818	100%	Preterm birth,LBW, Stillbirth	Low risk

### Prevalence of adverse fetal outcomes in Ethiopia

**Fig. 2:**
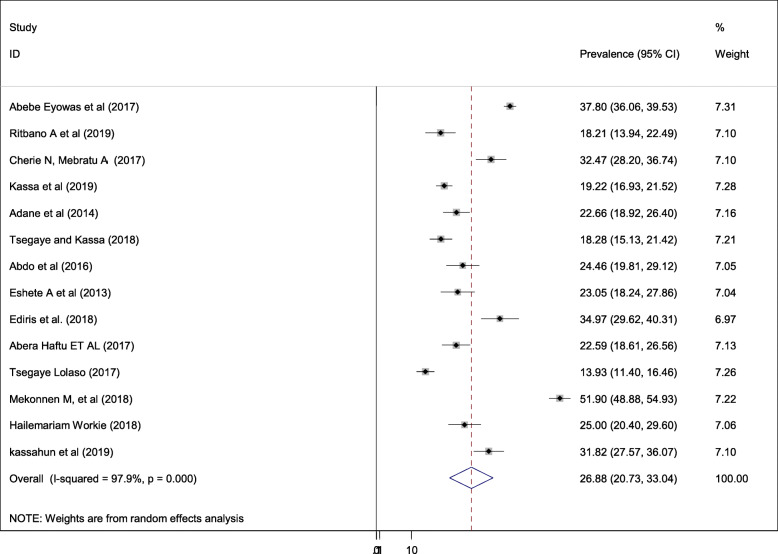
Forest plot of the overall pooled prevalence of adverse fetal outcomes in Ethiopia

This study is retrieved seventeen studies with a total population of 11,280 infants. The overall pooled prevalence of adverse fetal outcomes is presented with a forest plot **(**Fig. 2**).** Despite, the pooled estimated prevalence of adverse birth outcomes in Ethiopia was 26.88% (95% CI; 20.73–33.04; I^2^ = 97.9%, *P* < 0.001), the magnitude of each adverse neonatal outcomes is presented as follows; low birth weight (10.06%), preterm birth (8.76%), stillbirth (7.09%) and congenital anomalies accounted (2.55%).

### Pooled meta-analysis of different adverse fetal outcomes categories

#### Pooled prevalence of low birth weight

**Fig. 3:**
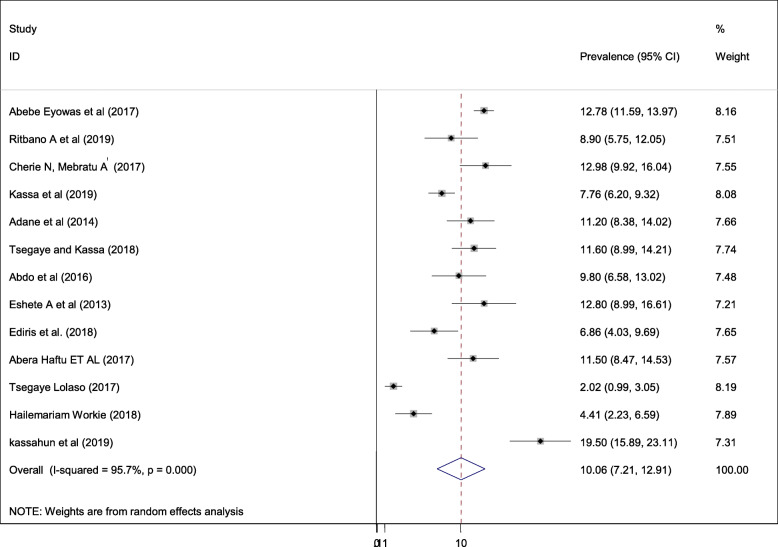
Forest plot of the pooled prevalence of adverse fetal outcomes (low birth weight) in Ethiopia

The quantified prevalence of low birth weight is presented in a forest plot **(**Fig. 3**).** The overall pooled prevalence of low birth weight was 10.06% (95% CI; 7.21–12.91; I2 = 95.7%, *p* < 0.001). In this systematic review and meta-analysis, the included studies were characterized by marked heterogeneity (I^2^ = 95.7%; *p* < 0.001). Furthermore, no publication bias was detected using Egger’s tests with a p-value of 0.091.

#### Pooled prevalence of preterm birth

**Fig. 4:**
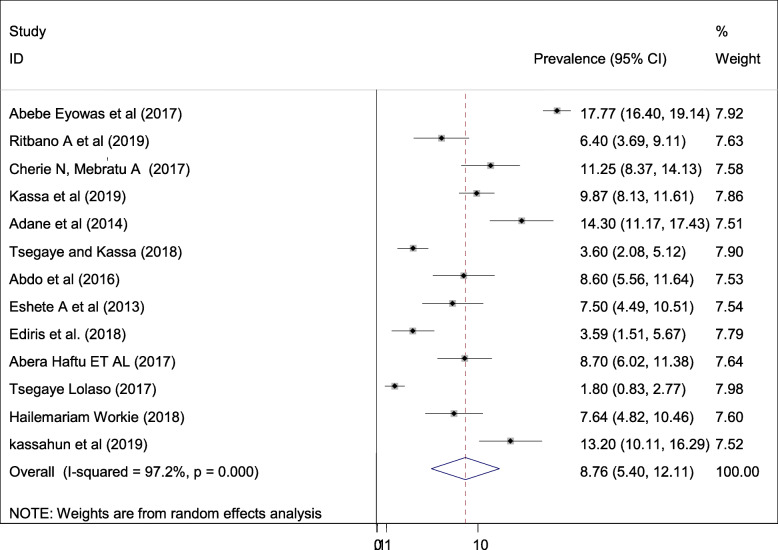
Forest plot of the pooled prevalence of adverse fetal outcomes (prematurity) in Ethiopia

The estimated prevalence of preterm birth is presented in a forest plot **(**Fig. 4**).** The overall pooled prevalence of prematurity was 8.76% (95% CI; 5.4–12.11; I^2^ = 97.2%, *p* < 0.001). In this systematic review and meta-analysis, the included studies were characterized by marked heterogeneity (I^2^ = 97.2%; *p* < 0.001). Furthermore, no publication bias was detected using Egger’s tests with a *p*-value of 0.26.

#### Pooled prevalence of stillbirth

**Fig. 5:**
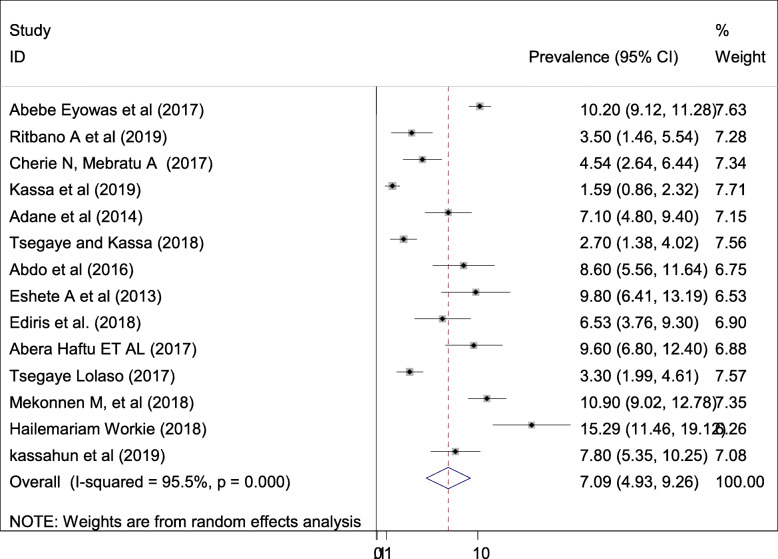
Forest plot of the pooled prevalence of adverse fetal outcomes (still birth) in Ethiopia

The estimated prevalence of fetal death is presented in a forest plot **(**Fig. 5**).** The overall pooled prevalence of fetal death was 7. 09% (95% CI; 4.93–9.26; I^2^ = 95.5%, *p* < 0.001). In this systematic review and meta-analysis, the included studies were characterized by marked heterogeneity (I^2^ = 95.5%, *p* < 0.001). Furthermore, low publication bias was detected using Egger’s tests with a *p*-value of 0.03.

### Pooled prevalence of congenital defect

**Fig. 6:**
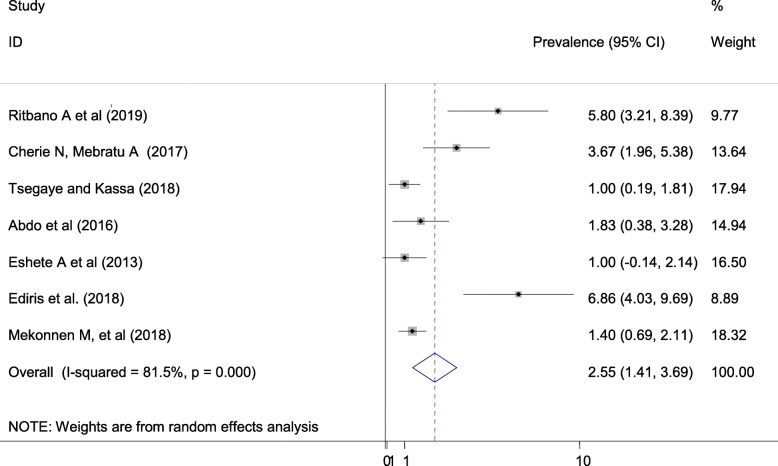
Forest plot of the pooled prevalence of adverse fetal outcomes (congenital defect) in Ethiopia

The estimated prevalence of congenital defects is presented in a forest plot **(**Fig. 6**).** The overall pooled prevalence of congenital defect was 2.55% (95% CI; 1.41–3.69; I^2^ = 81.5%, *p* < 0.001). In this systematic review and meta-analysis, the included studies were characterized by marked heterogeneity (I^2^ = 81.5%, *p* < 0.001). Furthermore, possibility of publication bias was detected using Egger’s tests with a *p*-value of 0.006.

### Publication bias

**Fig. 7:**
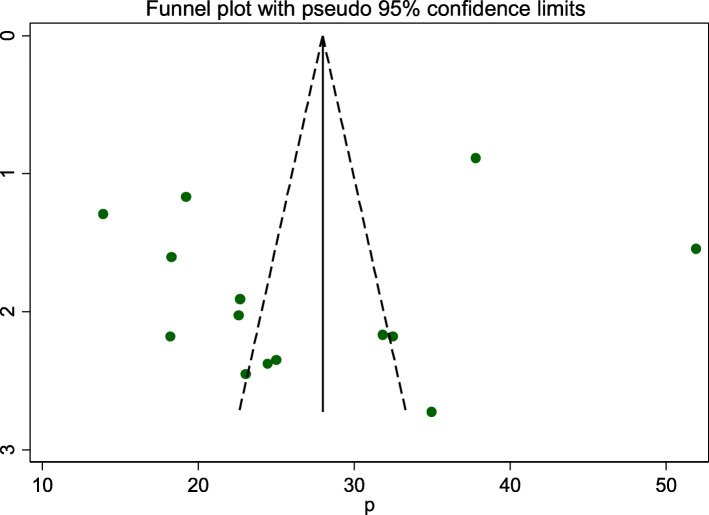
Funnel plot to show publication bias

A funnel plot was assessed for asymmetry distribution of adverse fetal outcomes by visual inspection **(**Fig. 7**).** Egger’s regression test showed with a p-value of 0.522 showed that the absence of publication bias.

### Subgroup analysis

Subgroup analysis was employed with the evidence of heterogeneity. Hence the Cochrane I^2^ statistic =97.9%, *P* < 0.001) with evidence of marked heterogeneity. Therefore subgroup analysis was done by publication year and study area **(**Table 2**).**

**Table 2:** Sub group analysis on the prevalence of adverse fetal outcomes in Ethiopia

Variables	subgroup	No. of studies	Model	Prevalence (95%CI)	I^2^ (%)	*P* value
Publication year	2013–2016	3	random	23.3(20.8, 25.8)	97.9	< 0.001
	2016–2019	11	random	27.83(20.4–35.3)	98.4	< 0.001
Study area	SNNPR	4	random	18.4 (14.3, 22.6)	81.7	< 0.001
	Amhara	6	random	27.87 (20.2, 35.6)	97.3	< 0.001
	Tigray	2	Fixed	23.6 (20.6, 26.66)	0	0.437
	Others^a^	2	Random	43.6(27, 60.2)	96.6	< 0.001

Others^a^ (Oromia and Somali)

### Associated factors for adverse fetal outcomes

In this systematic review and meta-analysis; rural residency, lack of antenatal care follow up, pregnancy, current pregnancy complication, and advanced maternal age were the factors for adverse fetal outcomes **(**Table 3**).**

**Table 3:** Summary of associated risk factors for adverse birth outcome in Ethiopia

Variables	Model	Publication bias Egger test	Status of heterogeneity	AOR(95%CI)	I^2^ (%)	*P* value
Rural residency	Random	0.001	Low heterogeneity	2.31(1.64, 3.24)	32.1	0.183
Current pregnancy complication	Random	0.952	moderate heterogeneity	4.98 (2.24, 11.07)	85.9	< 0.001
Not having antenatal care	Random	0.136	Low heterogeneity	3.84 (2.76,5.35)	23.1	0.238
Advanced maternal age ≥ 35	Random	0.008	Moderate heterogeneity	2.72 (1.62, 4.58)	55.6	0.08
Pregnancy induced-hypertension	Fixed	0.065	No heterogeneity	7.27 (3.95, 13.39)	0	0.868

Women who were in rural residency (AOR = 2.31; 95% CI: 1.64–3.24) 2.3 times more likely to have adverse fetal outcomes than women who were living in an urban area.

Women who hadn’t antenatal care follow up (AOR = 3.84; 95% CI: 2.76–5.35) 3.84 times more likely to have adverse fetal outcome than women who had antenatal care follow up.

Women who had current pregnancy complications (AOR = 4.98; 95% CI: 2.24–11.07) nearly 5 times more likely to have adverse fetal outcome than women who hadn’t current pregnancy complication.

The odds of having advanced maternal age ≥ 35 (AOR = 2.72; 95% CI: 1.62–4.58), had a high chance of developing adverse fetal outcome.

In this study women who had pregnancy-induced hypertension (AOR = 7.27; 95% CI: 3.95–13.39), were 7.27 times more likely to develop adverse fetal outcomes than their counterparts.

## Discussion

Pregnancy outcomes in low- and many middle-income countries are far worse than those in high-income countries. In this systematic review and Meta-analysis, the pooled prevalence of adverse fetal outcomes in Ethiopia was 26.88% (95% CI; 20.73–33.04). The most common adverse birth outcome categories were low birth weight of 10.06% (7.21–12.91), and preterm birth 8.76% (5.4–12.11).

This meta-analysis was estimated the national prevalence of stillbirth among adverse fetal outcomes in Ethiopia. Hence, the overall pooled prevalence of stillbirth was 7. 09% (4.93–9.26). This review finding is lower than the study conducted in India [25.3%], Pakistan [56.9%] and Guatemala [19.9%] [38, 39]. This discrepancy might be due to the study participants included in this systematic review and meta-analysis were reviewing in a single country with multiple original studies; might have lower representatives as compared to studies conducted at a global level consisting of many countries at a point.

This review was estimated the overall prevalence of preterm (prematurity) among adverse fetal outcomes in Ethiopia. Hence, the overall pooled prevalence of preterm was 8.76% (5.4–12.11). This study finding is in line with the study done in Asia [10.4%], North America [11.2%], Sub-Saharan Africa [12%], Nigeria [11.4%], South Korea [7.1%], Nebraska [5.54%] and Indonesia [10.4%] [40–42]. This finding is consistent with Asian and African countries, because of the health package and health care system towards maternal and neonatal health is nearly similar. Besides, now a day’s countries are implementing different strategies and preventive modalities to preterm birth in collaboration with governmental and non-governmental organizations for African countries including Ethiopia, as a result, this finding is lower as compared to the WHO target level of prematurity for the contribution of neonatal mortality and morbidity.

This review was estimated the overall prevalence of congenital defects among adverse fetal outcomes in Ethiopia. Hence, the overall pooled prevalence of congenital defects was 2.55% (2.41–3.69). This review finding is lower than the study done in sub-Saharan Africa [20%], and Nigeria [6.3%] [43, 44]. The discrepancy of these study findings may be due to the association of the participants in terms of different characteristics; such as residence, socio-demographic factors, behavioral factors, genetic factors, environmental factors, and socioeconomic status. Besides, iron-folic acid supplementation during pregnancy is decreasing the congenital anomalies by 70%, therefore, in Ethiopia, the supplementation is highly practicing and implementing now a day’s widely.

This review was estimated the overall prevalence of low birth weight among adverse birth outcomes in Ethiopia. Hence, the overall pooled prevalence of low birth weight was 10.06% (7.21–12.91). This study finding is in line with the study done in Indonesia [12.9%], Armenia [9.0%, higher than the study conducted in Nigeria [6.3%] and lower than the study done in Kenya [12.3%], Tanzania (13.9%), South Africa [38.54%] [45–48]. Low birth weight has different known and idiopathic risk factors; such as environmental and lifestyle risk factors, fetal risk factors, obstetric related factors, medical-related factors, and maternal & family socio-demographic risk factors. Having the supremacy of the above-motioned risk factors in each country may be increasing the magnitude of the preterm birth even death may have happened secondary to prematurity of the baby.

The odds of having advanced maternal age ≥ 35 years nearly three times to have adverse fetal outcomes. This finding is in line with the study done in Cameroon [49], low income countries [39], India [50], Nigeria [51], African lake regions [52], Uganda [53]. This might be due to the age of the women directly linked with parity. Therefore, high parity women at risk for developing different labor and delivery complications that lead to both fetal and maternal outcomes due to the laxity of the uterus in repeated and short inter interval pregnancy.

The odds of having pregnancy-induced hypertension were nearly three times to have adverse birth outcomes. This finding is in line with the study done in Cameroon [49], Kenya [54], India [50], Nigeria [51], and Bangladesh [55]. This might be due to endothelial cell injury and vasoconstriction of blood vessels which causes placental insufficiency.

Pregnancy complication is one of the associated risk factors in this meta-analysis. Having pregnancy complications was almost five times more likely to develop adverse fetal outcomes. This study finding is in line with the study done in Bangladesh [55]. Brazil [56], Kenya [57] and China [58]. The possible reason might be due to women who have current pregnancy complications such as; premature rupture of membrane, antepartum hemorrhage, and abnormal labor and pregnancy are the most common pregnancy and labor complications that cause preterm birth, stillbirth, and low birth weight.

Lack of antenatal care follow up is the associated risk factor for adverse fetal outcomes in this meta-analysis. Having no ANC follow up was four times more likely to develop adverse birth outcomes. This study finding is in line with the study done in Tanzania [59], and Gambia [60]. This might be due to During ANC follow up women will have a chance to access information related to danger signs of pregnancy. Having regular ANC follow up will also help a pregnant woman seek early treatment for her potential pregnancy-related problems [27].

The odds of living in rural were two times more likely to develop adverse fetal outcomes. This study finding is in line with the study done in China [61], and Brazil [62]. This might be due to women who live in rural areas aren’t getting health care services comprehensively and they are less likely to be informed about the danger sign and complication of pregnancy, labor, and delivery. Furthermore, cultural behaviors in rural areas have a great effect on the nutritional status of women through the prohibition of essential foods and or drinks [36]. Publication bias has happened if one or more of the following has existed: selection bias, true heterogeneity, artifact, and chances are the main sources of publication bias. Large studies are likely to be published regardless of statistical significance because these studies involve large commitments of time and resources whereas Small studies are at greatest risk for being lost, because of the small sample size. In this study publication bias was not detected (Eggers, *p* value = 0.522) of the polled estimated prevalence of adverse fetal outcomes.

### Limitations of the study

Including papers only published by the English language and accessing only hospital-based studies was the restraint of the study. It might lack national representativeness because no data were from all regions.

## Conclusion

In this study, the overall pooled prevalence of adverse fetal outcomes in Ethiopia was high. Rural in residency, lack of antenatal care follow up, pregnancy-induced hypertension, advanced maternal age ≥ 35, and having a current complication of pregnancy were the factors associated with adverse fetal outcomes. Therefore, based on the study findings, the authors recommend particular emphasis shall be given to have regular antenatal care follow up, health education, early detection, and intervention of obstetric complications. Creating awareness of women on the effect of pregnancy at an advanced age, and providing timely and focused antenatal care (ANC) follow up to all pregnant women are very crucial to reduce the magnitude of the problem.

## Data Availability

All related data has been presented within the manuscript. The dataset supporting the conclusions of this article is available from the authors on request.

## Abbreviations

CI: Confidence Interval
EDHS: Ethiopian demographic and health survey
EMDHS: Ethiopian Mini demographic and health survey
LBW: Low Birth Weight
OR: Odd Ratio
SNNPR: South Nation Nationalities and Peoples region
